# Relationship between the stuttering severity index and speech rate

**DOI:** 10.1590/S1516-31802003000200010

**Published:** 2003-03-05

**Authors:** Claudia Regina Furquim de Andrade, Luciana Maluf Cervone, Fernanda Chiarion Sassi

**Keywords:** Stuttering, Speech, Rate, Diagnosis, Gagueira, Velocidade de Fala, Diagnóstico

## Abstract

**CONTEXT:**

The speech rate is one of the parameters considered when investigating speech fluency and is an important variable in the assessment of individuals with communication complaints.

**OBJECTIVE:**

To correlate the stuttering severity index with one of the indices used for assessing fluency/speech rate.

**DESIGN:**

Cross-sectional study.

**SETTING:**

Fluency and Fluency Disorders Investigation Laborator y, Faculdade de Medicina da Universidade de São Paulo.

**PARTICIPANTS:**

Seventy adults with stuttering diagnosis.

**MAIN MEASUREMENTS:**

A speech sample from each participant containing at least 200 fluent syllables was videotaped and analyzed according to a stuttering severity index test and speech rate parameters.

**RESULTS:**

The results obtained in this study indicate that the stuttering severity and the speech rate present significant variation, i.e., the more severe the stuttering is, the lower the speech rate in words and syllables per minute.

**DISCUSSION AND CONCLUSION:**

The results suggest that speech rate is an important indicator of fluency levels and should be incorporated in the assessment and treatment of stuttering. This study represents a first attempt to identify the possible subtypes of developmental stuttering.

**DEFINITION:**

Objective tests that quantify diseases are important in their diagnosis, treatment and prognosis.

## RESUMO

**CONTEXTO:**

A velocidade de fala é um dos parâmetros analisados na fluência da fala e é considerada uma variável importante na avaliação de indivíduos com queixa de comunicação.

**OBJETIVO:**

Relacionar a gravidade da gagueira com um dos índices utilizados para avaliar fluência/velocidade de fala.

**TIPO DE ESTUDO:**

Transversal.

**LOCAL:**

Laboratório de Fluência e Desordens da Fala da Faculdade de Medicina, Universidade de São Paulo.

**PARTICIPANTES:**

70 adultos com diagnóstico de gagueira.

**VARIÁVEIS ESTUDADAS:**

Uma amostra de fala contendo no mínimo 200 sílabas expressas foi audiofilmada de cada um dos participantes e analisada segundo teste objetivo para determinação da gravidade da gagueira e segundo os parâmetros de análise de velocidade de fala.

**RESULTADOS:**

Os resultados obtidos no estudo indicam que a gravidade da gagueira e a velocidade de fala apresentam variação significante, ou seja, quanto mais gravea gagueira, menor a velocidade de fala em palavras e sílabas por minuto.

**DISCUSSÃO E CONCLUSÃO:**

Os resultados sugerem que a velocidade de fala é um indicador importante dos níveis de fluência e deve ser incorporada nas avaliações e tratamentos de gagueira. Esse estudo representa um primeiro esforço para identificação de possíveis subtipos de gagueira desenvolvimental. Testes objetivos que quantificam as doenças são importantes para a definição do diagnóstico, do tratamento e do prognóstico.

## INTRODUCTION

Fluent speech is the ability to talk with continuity, at a sustained rate and without effort.^1,2^ The level of fluency seems to vary from one individual to the next and within the same individual, depending on the day, the emotions, the mastery of a given conversation subject and the different communication situations within everyday life.^2^ The level of fluency or dysfluency can be assessed according to different parameters (typology of speech disruptions, frequency of speech disruptions, speech rate, latency and associated movements).

The speech rate is one of the parameters analyzed when investigating speech fluency and is an important variable in the assessment of individuals with communication complaints.^3^ According to the literature, speech rate is also an important index when analyzing the effectiveness of treatment, since one of the goals of speech therapy is to provide for the patient the ability to present the same speech pattern as that of individuals with no communication deficits, i.e. such that the patient's speech does not sound different from that of fluent speakers.

The speech rate is a significant tool for the understanding of time control in normal development, as well as for the identification and manipulation of time differences in the processing of speech disorders.^4^ Information relating to determining the most effective type of treatment for each patient also can be obtained using the parameter of speech rate.

Some authors^5^ believe that there are two main theories related to speech rate and stuttering. The first is the psycholinguistic model, in which child and adult stutterers require more time to process linguistic and phono-logical information. Because of this delay, the level of fluency in stutterers is lower than in fluent speakers. The second theory is to consider stuttering as a neuromotor and rhythmic disorder that is linked to the rate of articulation and reflects the control of compensatory movements.

Speech rate can be measured not only in words per minute, which indicates the rate at which information is produced, but also in syllables per minute, which indicates the rate of articulation, that is, the rate at which the structures involved in the production of speech are modified.^2^ Findings from a study developed among fluent adult speakers of the Brazilian Portuguese language have indicated that the normal speech rate is in the range of around 218.8 to 256.5 syllables per minute and 117.3 to 140.3 words per minute.^6^

The present study aims to relate the stuttering severity to the speech rate, gender and age.

## METHODS

### Subjects

Seventy adults (aged 18 years and/or older) participated in this study, of whom 51 were male and 19 were female, with no racial distinction. All were native speakers of the Brazilian Portuguese language and had been diagnosed as stutterers, varying in literacy and with no other communication or health deficits.

All patients were seen at the Fluency and Fluency Disorders Investigation Laboratory of the Speech-Language and Hearing Pathology Division of the University of São Paulo.

All speech samples were recorded using a video camera (Panasonic-NV-VJ98PN), which was fixed on a tripod. For the analysis of the stuttering severity, an international instrument was adopted (Stuttering Severity Instrument^7^) and for the analysis of the speech rate, a specific protocol was used.^8^

The study had prior approval from the Research Ethics Committee of the Department of Physiotherapy, Speech-Language and Hearing Pathology and Occupational Therapy (n^°^ 02/223), and informed consent was obtained from all patients.

### Procedures

All of the participants underwent a complete speech and language evaluation. Participants who were diagnosed as having other speech and/or language deficits in association with stuttering were excluded from the research.

Speech samples were obtained in a situation of spontaneous speech (visual stimulus). Self-expressive speech is that which does not require attention to any of the production aspects besides those involved in the generation of the linguistic message.^9^ It expresses the feelings and intentions of the speaker, formulated in a linguistic code – phonological, syntactic, semantic and pragmatic – with communicative intention.

Each speech sample was recorded on a video camera and contained at least 200 fluent syllables. The samples were transcribed literally.^8^

The stuttering severity index and the speech rate indices (words and syllables per minute) were obtained using the criteria described below:

- Stuttering Severity Instrument for Children and Adults^7^ – This test assesses the frequency and duration of speech disruptions, as well as the presence of physical concomitants associated with these disruptions. Based on these parameters, the stuttering severity index is determined as very mild, mild, moderate, severe or very severe.
- Speech rate^2,8^ – This test determines the number of words and syllables per minute. The number of words per minute indicates the index of information production in speech. The number of syllables per minute indicates the articulation rate index, i.e. it indicates the motor transition ability.

## RESULTS

The data obtained are presented in the form of a table and graphs. Table 1 illustrates the relationship between the stuttering severity index and the speech rate (words and syllables per minute).

**Table 1:** Variance between the stuttering severity index and speech rate (words/minute; syllables/minute)

Variation source	Degrees of freedom	Mean square	F	Significance of F
Words/min	4	4201.02	6.145	< 0.001*
Error	65	683.61		
Syllables/min	4	11907.18	4.588	0.003*
				
Error	65	2595.03		

* *= statistically significant;*

*F = ratio between the average of the samples and the average of the sample variance*

It is important to highlight that for diagnostic purposes an additional stuttering severity index not found in the American test was used: normal with a complaint. For these individuals (n = 9) the American test was not sensitive, i.e. they were classified as normal. Since these adults were looking for stuttering treatment and showed pertinent symptomatology (short duration speech disruptions, therefore not punctuated by the stuttering severity instrument), these individuals were included in the present research.

**Figure 1:**
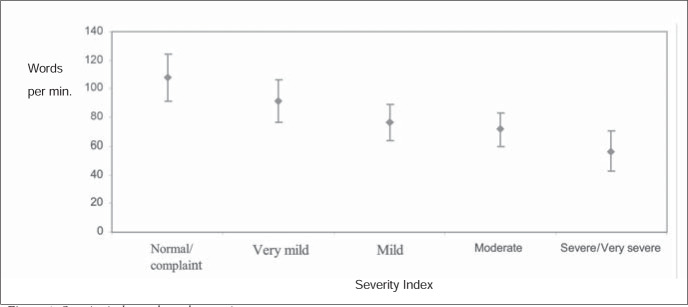
Severity index and words per minute.

**Figure 2:**
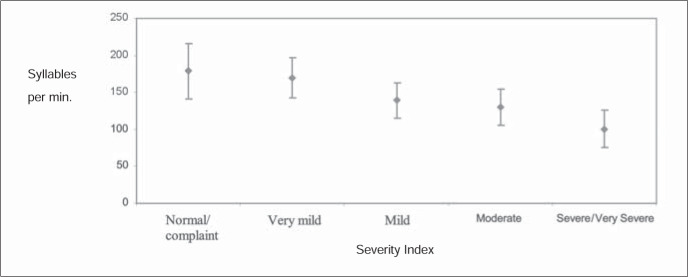
Severity index and syllables per minute.

The statistical test used was analysis of variance (ANOVA), with a significance level of 5% (confidence interval for the average ± 1.96 standard deviation/root). The analysis of the results indicated that there was a statistically significant difference for the relationship investigated, as illustrated by Figures 1 and 2.

With regard to the variation in words per minute (the index of speech production), as illustrated by Figure 1, the difference between the stuttering severity indices indicate that the more severe the stuttering was, the smaller the number of words produced per minute was, thus tracing out a gradual decrease. For example, in a very mild stuttering the mean number of words per minute is 80.61 and in a very severe stuttering this number is 44.98.

For the variation of syllables per minute (the index of articulation transition ability) (Figure 2), the difference between the stuttering severity indices also indicated progressive decrease, i.e. the more severe the stuttering was, the lower the articulation ability was. For example, in a very mild stuttering the mean number of syllables per minute is 146.31 and in a very severe stuttering this number is 80.77.

**Figure 3:**
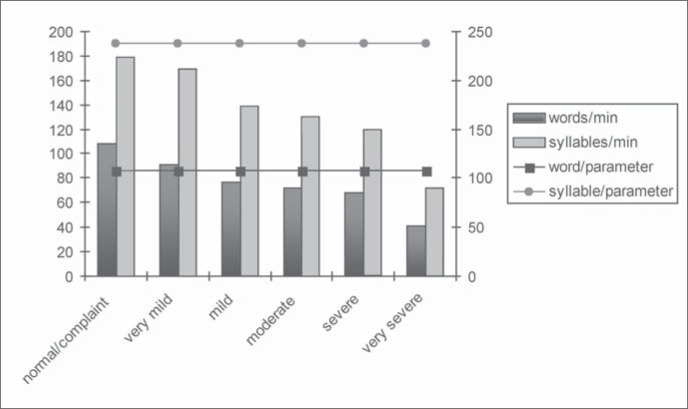
Severity index profile and speech rate.

Figure 3 presents a graph comparing the stuttering severity index of the participants in this study with the indices of speech rate for fluent Brazilian adults accordingly to a measurement made by Zackiewicz and Andrade.^10^

## DISCUSSION

Despite the behavioral complexity of a full stutter problem, dysfluency often plays a primary role in differential diagnostic decisions and treatment evaluations. It is known that absolute continuity of speech production is physiologically impossible. The perception of continuous speech can be obtained by the number of audible speech utterances and by the reduction in the duration of the physiological pauses (e.g. intervals for swallowing and breathing) and linguistic pauses (e.g. memory effects and lexical access) that are pertinent and expected for any speaker.^11-13^

The neurophysiological processing of speech fluency depends on the stability of temporal coordination between the motor execution abilities and the performed cognitive processing. Developmental stuttering presents as a chronic disruption in an individual's ability to produce smooth, effortless, and forward-moving speech. Results from behavioral genetic studies performed over the past two decades have uniformly implicated genetic factors in the etiology of developmental stuttering.^9,14-21^

Brain imaging by positron emisson tomography (PET) or single-photon emission computed tomography (SPECT) studies of stutter probands have already been successful in identifying candidate regions of interest that distinguish affected from unaffected individuals. So far, these studies have indicated inter-hemispheric functional asymmetry, i.e. in fluent speakers the activation for speech and language is predominantly from the left hemisphere, whereas in individuals who stutter, this activation is diffuse or predominantly from the right hemisphere. Permanent hypometabolism of the left caudate has also been identified, and it has been observed that in individuals who stutter this basal ganglion is almost 50% less active than in fluent speakers. Apparently there is a decrease in the activity of the cerebellum circuit components in individuals who stutter, in comparison with fluent individuals. This is aggravated when using spontaneous speech, whereas this activity becomes normal in a situation of induced fluent speech (e.g. reading in chorus). When compared to fluent individuals, stutterers demonstrate cortical hypoactivity of the areas associated with language processing (Broca) and hyperactivity of the areas associated with motor functions.^22-30^

The study here presented has confirmed the findings previously published about speakers of the American English language, pointing to a direct relationship between rises in the stuttering severity index and reductions in speech rate, not only for the information production but also for the articulation transition.^31-35^

In Brazil, the evaluation of stuttering is usually indirect, perceptual, and based exclusively on the patient's complaint or the professional's judgement. Objective tests that quantify the pathology are important for the diagnosis, treatment and prognosis.^36-41^ Some of the variables that are known to complicate the molecular study of complex disorders, including stuttering, are: absence of diagnostic standards (different criteria for determining affected status); variable expression (individuals may present with very mild or sub-clinical variants of a disorder); absence of accepted analysis strategies for finding multiple susceptibility genes (techniques and strategies for identifying multiple susceptibility loci that interact to produce a pathological condition); and replication problems (a pervasive problem in genetically complex disorders, especially regarding the influence of the originally spoken language in the speech and language processing).^42^

Finally, this study represents a first attempt to identify the possible subtypes of developmental stuttering. With the establishment of more objective and more precise diagnostic criteria, it will be possible to structure the basis for a classification system that will reduce subject heterogeneity and allow a positive progress in genetic analysis.

## CONCLUSION

The results suggest that speech rate is an important indicator of fluency levels and should be incorporated in the assessment and treatment of stuttering. This study represents a first attempt to identify the possible subtypes of developmental stuttering.
